# The European Heritage of Folk Medicines and Medicinal Foods: Its Contribution to the CAMs of Tomorrow

**DOI:** 10.1155/2013/827521

**Published:** 2013-04-09

**Authors:** Andrea Pieroni, Manuel Pardo-de-Santayana, Fabio Firenzuoli, Cassandra L. Quave

**Affiliations:** ^1^University of Gastronomic Sciences, Piazza Vittorio Emanuele 9, Pollenzo, 12060 Bra, Italy; ^2^Departamento de Biología (Botánica), Universidad Autónoma de Madrid, c/Darwin 2, Campus de Cantoblanco, 28049 Madrid, Spain; ^3^Center for Integrative Medicine, Careggi and Department of Preclinical and Clinical Pharmacology, University Hospital of Florence and “M. Aiazzi Mancini”, University of Florence, Viale G. Pieraccini 6, 50139 Florence, Italy; ^4^Center for the Study of Human Health, Emory University, 550 Asbury Circle, Candler Library 107, Atlanta, GA 30322, USA

Europe has shared a vibrant history in the use of herbal medicines since ancient times. Still today, it serves as a crucial hotspot for the production and utilization of herbal remedies. Despite the popularity of homemade and commercial herbal products in Europe, a key disconnect still exists in the dialog between allopathic practitioners and patients that use CAM herbal products. There is a pressing need to connect the evidence-based use of medicinal plants and their derivatives to emerging strategies of medicine and conceptual models of healing, which aim to be culturally sensitive in nature and take into account the cultural and historical backgrounds of patients. A key turning point in this challenge has come from the interdisciplinary science of medical ethnobiology, which addresses traditional/folk uses of plants and other remedies, in both rural and urban environments. In this special issue of five papers, our aim was to showcase some of the challenges and advancements made in this field as illustrated through case studies of medico-ethnobotanical research and recent findings in the study of folk phytopharmaceuticals, while also addressing some of the most crucial bottlenecks limiting their transition into clinical practice. 

The guiding paper of this special issue “*Medical ethnobotany in Europe: from field ethnography to a more culturally sensitive evidence-based CAM*?” provides a review of the medico-ethnobotanical field studies conducted in Europe over the past two decades, which have been focused primarily in the South and South-Eastern regions of rural Europe. This review demonstrates that such field studies represent an important foundation for understanding local small-scale uses of CAM natural products and allows for the assessment of how certain products could potentially be expanded into the global market. The article also delineates how field studies of this nature can provide useful information to the allopathic medical community as they seek to reconcile existing and emerging CAM therapies with conventional biomedicine. Some of the problems related to the status of traditional medical knowledge, which is at risk due to acculturation trends, are also addressed with the goal of fostering a sense of urgency to document and conserve knowledge concerning folk uses of medicinal plants.

In the second article “*Plant ethnoveterinary practices in two Pyrenean territories of Catalonia (Iberian Peninsula) and in two areas of the Balearic islands and comparison with ethnobotanical uses in human medicine*,” the authors present the results of an ethnobotanical study focused on veterinary uses of plants in two Catalan Pyrenean and two Balearic regions, where approximately one hundred of plant taxa have been claimed to be useful for such purposes. A significant number of the plants discussed here have never been previously reported as medicinal. Moreover, the authors demonstrate how several ethnoveterinary applications coincide with those in human medicine, thus illustrating how a community's conceptual *herbal landscape* is the result of a complex ecological eco-evolution, where humans, animals, and plants are intricately linked.

Likewise, a similar topic is assessed in the third paper “*The relationship between plants used to sustain finches (Fringillidae) and uses for human medicine in Southeast Spain*,” in which the authors describe their study of the complex interplay between local medicinal plants and plants traditionally used by wild bird hunters and breeders to capture and promote the captive breeding of songbirds in Southern Spain.

A fourth article “*Comparative medical ethnobotany of the senegalese community living in Turin (Northwestern Italy) and in Adeane (Southern Senegal)*” serves as an example of a case study on migrant health strategies in Europe. Here, the authors describe a medico-ethnobotanical survey conducted among both healers and laypeople living in one Senegalese migrant community of Northern Italy as it compares to the ethnomedical practices of their peers living in Adeane (Southern Senegal). This study reports that the large majority of the medicinal plants recorded among Senegalese migrants were also used in their country of origin, thus demonstrating the resilience of home remedies among migrants in Europe. Importantly, the authors also discuss the potential role that such data could have in shaping public health policies devoted to migrant groups in Western Countries, which seek to seriously take into account culturally sensitive approaches in the form of emic health-seeking strategies.

In the last article of the series “*Can estragole in fennel seed decoctions really be considered a danger for human health? a fennel safety update*,” the authors discuss their investigation of the safety of one of the most widely used European medicinal plants and flavoring agents in food products: fennel. The safety of herbal products is a top concern for their transition into mainstream use. Recently, due to reports of estragole carcinogenicity, fennel has been alleged to be dangerous for humans—especially if used as decoction for babies. In this paper, the authors challenge these allegations, pointing out that estragole is inactivated by many substances contained in the decoction.

